# Cortical Measures of Phoneme-Level Speech Encoding Correlate with the Perceived Clarity of Natural Speech

**DOI:** 10.1523/ENEURO.0084-18.2018

**Published:** 2018-04-16

**Authors:** Giovanni M. Di Liberto, Michael J. Crosse, Edmund C. Lalor

**Affiliations:** 1 School of Engineering, Trinity Centre for Bioengineering, and Trinity College Institute of Neuroscience, Trinity College Dublin, Dublin 2, Ireland; 2 Department of Pediatrics and Department of Neuroscience, Albert Einstein College of Medicine, Bronx, New York 10461; 3 Department of Biomedical Engineering and Department of Neuroscience, University of Rochester, Rochester, New York 14627

**Keywords:** cortical entrainment, EEG, intelligibility, phonetics, predictive coding, prior knowledge

## Abstract

In real-world environments, humans comprehend speech by actively integrating prior knowledge (P) and expectations with sensory input. Recent studies have revealed effects of prior information in temporal and frontal cortical areas and have suggested that these effects are underpinned by enhanced encoding of speech-specific features, rather than a broad enhancement or suppression of cortical activity. However, in terms of the specific hierarchical stages of processing involved in speech comprehension, the effects of integrating bottom-up sensory responses and top-down predictions are still unclear. In addition, it is unclear whether the predictability that comes with prior information may differentially affect speech encoding relative to the perceptual enhancement that comes with that prediction. One way to investigate these issues is through examining the impact of P on indices of cortical tracking of continuous speech features. Here, we did this by presenting participants with degraded speech sentences that either were or were not preceded by a clear recording of the same sentences while recording non-invasive electroencephalography (EEG). We assessed the impact of prior information on an isolated index of cortical tracking that reflected phoneme-level processing. Our findings suggest the possibility that prior information affects the early encoding of natural speech in a dual manner. Firstly, the availability of prior information, as hypothesized, enhanced the perceived clarity of degraded speech, which was positively correlated with changes in phoneme-level encoding across subjects. In addition, P induced an overall reduction of this cortical measure, which we interpret as resulting from the increase in predictability.

## Significance Statement

The human ability to comprehend speech despite challenges such as loud noise and competing speech derives in large part from the use of prior knowledge (P) of the upcoming speech. Here, we examine the cortical underpinnings of this process by using P to modulate the perceived intelligibility of degraded stimuli. We find two distinct effects of P: a positive correlation between perceptual enhancement and phoneme-level encoding and an overall suppression of this cortical encoding.

## Introduction

Successful speech comprehension in noisy, real-world environments is conducted by a complex hierarchical system in the human brain (Chang et al., 2010; Okada et al., 2010; Peelle et al., 2010; DeWitt and Rauschecker, 2012; Hickok, 2015). In such cases, it is widely acknowledged that an active cognitive process takes place where speech perception is strongly influenced by prior knowledge (P) and a contextual expectation of upcoming speech input (McClelland and Elman, 1986; Davis and Johnsrude, 2007; McClelland, 2013; Heald and Nusbaum, 2014; Leonard and Chang, 2014). However, the nature of this influence is not yet well understood.

Firstly, it remains unclear at what hierarchical processing stages, and in particular how early, the encoding of speech is affected by top-down influence (Davis and Johnsrude, 2007). Studies using prior information to enhance the perception of degraded speech report that subjects experience a strong perceptual pop out effect whereby they report a marked increase in the perceived clarity of the speech as they process it in real time (Blank and Davis, 2016; Holdgraf et al., 2016; Tuennerhoff and Noppeney, 2016). This suggests that prior information might affect speech processing *in situ* in lower-level sensory processing areas at the acoustic and phonetic encoding stages, something that has been observed for effects such as phoneme restoration in noise (Leonard et al., 2016). However, event-related potential evidence on this issue has suggested that prior information first modulates activity in higher-order areas, which then feeds back to affect lower-level sensory processing at longer latencies (Sohoglu et al., 2012).

A second unresolved issue is the mechanism through which prior information affects bottom-up sensory processing. One view is that the neural encoding of a stimulus is enhanced by expectation (sharpening theories; McClelland and Elman, 1986; Mirman et al., 2006). An alternative theory, known as predictive coding, proposes that discrepancies (or errors) between what is predicted and what is received are passed from one level to the next within the speech processing hierarchy (Friston, 2005; Arnal and Giraud, 2012; Giraud and Poeppel, 2012). One recent functional magnetic resonance imaging (fMRI) study has provided strong evidence for a dominant role for predictive coding in the superior temporal sulcus (STS), by demonstrating interacting effects of prior expectation and sensory detail on multivoxel BOLD patterns (Blank and Davis, 2016). However, a recent study with invasive electrocorticography (ECoG) appeared to be more in line with the sharpening theory (Holdgraf et al., 2016). In particular, that study showed that P induces an enhancement of high-γ activity driven by rapid and automatic shifts in spectrotemporal tuning in auditory cortical areas. And the authors suggested that these shifts lead to changes in responsiveness to specific speech features, rather than a more general increase or decrease in activity (Holdgraf et al., 2016).

In this study, we aim to examine these two issues: (1) how early in the hierarchy is speech encoding affected by prior information; and (2) is the increase in perceived clarity that comes with prior information reflected in an enhancement or suppression of activity at particular hierarchical stages. To do this, we will use a recently introduced approach to EEG analysis that allows us to isolate early stage speech encoding with precise temporal resolution. The approach builds on the fact that dynamic cortical activity tracks the amplitude envelope of ongoing, natural speech (Aiken and Picton, 2008; Lalor and Foxe, 2010). It does so by assuming that this cortical speech tracking phenomenon reflects the activity of distinct neural populations that implement different functional roles (Ding and Simon, 2014). In particular, we seek to use forward encoding models to disambiguate contributions reflecting the processing of low-level speech acoustics from those reflecting the processing of categorical phonetic features (Mesgarani et al., 2014; Di Liberto et al., 2015). We aim to use this framework to analyze data collected during a perceptual pop-out speech experiment. Our primary hypothesis is that we will see a marked increase in the strength of the online encoding of phonetic features, in particular, between the cases where subjects hear unintelligible degraded speech versus when they can understand that same degraded speech as a result of having prior information.

## Materials and Methods

### Participants and data acquisition

Fourteen healthy subjects (eight males, aged between 21 and 31 years) participated in this study. Electroencephalographic (EEG) data were recorded from 128 electrode positions (plus two mastoid channels). Data were filtered over the range 0–134 Hz and digitized with a sampling frequency of 512 Hz using a BioSemi Active Two system. Monophonic audio stimuli were presented at a sampling rate of 44.1 kHz using Sennheiser HD650 headphones and Presentation software from Neurobehavioral Systems (http://www.neurobs.com). Testing was conducted in a dark room and subjects were instructed to maintain visual fixation on a crosshair centered on the screen, and to minimize motor activities for the duration of each trial. The study was undertaken in accordance with the Declaration of Helsinki and was approved by the Ethics Committee of the School of Psychology at Trinity College Dublin. Each subject provided written informed consent. Subjects reported no history of hearing impairment or neurologic disorder.

### Stimuli and experimental procedure

Audio-book versions of two classic works of fiction read in American English by the same male speaker were partitioned into 10-s speech snippets using MATLAB software (The MathWorks Inc.). A total of 120 snippets were randomly selected for the experiment. To alter the intelligibility of the speech, a method known as noise vocoding was implemented (Shannon et al., 1995; Davis and Johnsrude, 2003). This method filters the speech into a number of frequency bands and uses the amplitude envelope of each band to modulate band-limited noise. Specifically, the speech for this experiment was vocoded using three frequency bands logarithmically spaced between 70 and 5000 Hz according to Greenwood’s equation (70–494–1680–5000 Hz; Greenwood, 1961).

**Figure 1.**
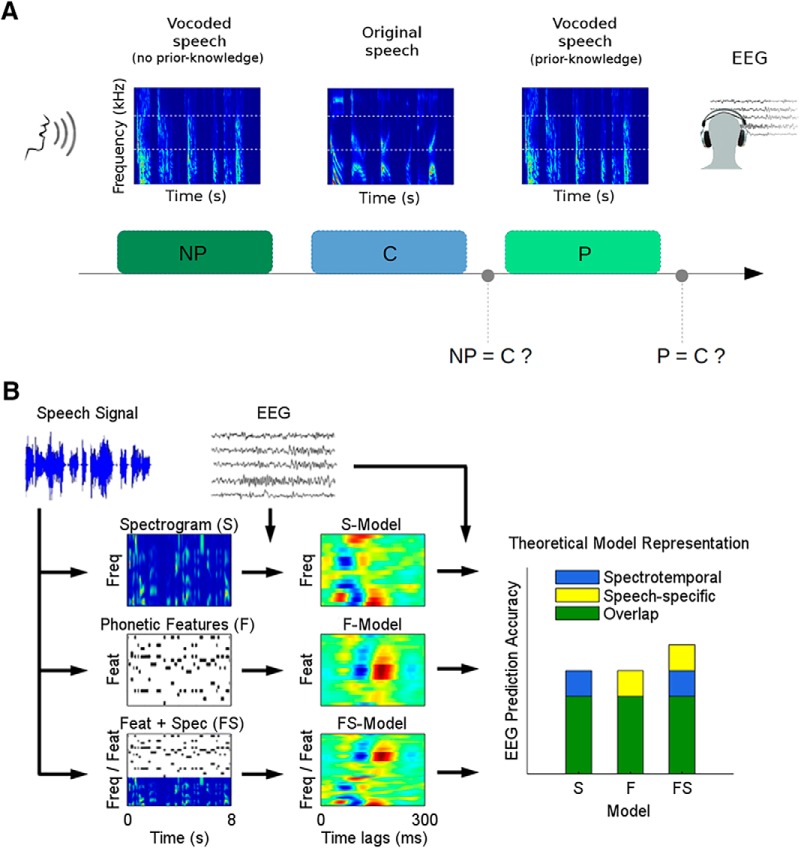
A pop-out experiment to modulate speech perception. ***A***, Experimental setup. EEG data were recorded while subjects listened to groups of three 10-s long speech snippets. In standard trials, the first (NP) and the third (P: prior knowledge) speech snippets were a three-channel noise-vocoded version of the second snippet (C: clear). In deviant trials, either the first or third snippets (or both) did not fully match the second snippet. After C and P, participants were asked to identify the first and the second vocoded snippets, respectively, as matching the clean speech or not (i.e., standard or deviant trial). ***B***, Analysis approach. A linear regression approach was used to derive mappings from different speech representations to the EEG. Regression models were fit for the acoustic spectrogram (S), a set of time-aligned phonetic features (F), and a combination of the two (FS). Each model was then tested for its ability to predict the EEG using leave-one-out cross-validation.

Each EEG standard trial consisted of the presentation of three speech segments (Fig. 1*A*). The first segment [no P (NP)] was degraded using noise vocoding; the second one [clear (C)] was the same 10-s speech segment, but in its original clear form; and the third presentation (P) was the noise-vocoded version again. As such, the first (NP) and third (P) speech segments involved identical acoustic stimuli, but it was hoped that the perceived clarity of the third segment (P) would be improved by the prior information provided by the interleaved segment C (perceptual pop-out effect). As a control measure, we also included deviant trials. These trials consisted of a modified version of NP and/or P, where a random chunk of ∼5 s was replaced with words from a different trial. For both NP and P, the probability of a deviant stimulus was set to 10%.

Participants were asked to make two judgements based on the stimuli. First, after presentation of segment C, they were asked to decide whether the first vocoded segment, NP, was deviant (different from C) or standard (the same as C). And second, after presentation of the second vocoded segment, P, they were asked to decide whether it was a deviant (different from C) or standard (the same as C). More specifically, they were asked to make both of these decisions using a level of confidence from 1 to 5 (“definitely a deviant,” “probably a deviant,” “I don’t know,” “probably a standard,” and “definitely a standard”). For standard trials, a higher confidence level when comparing segments P and C than when comparing segments NP and C was taken as evidence of enhanced perceived speech clarity. This score was normalized by subtracting a subject-specific baseline that was obtained by performing the same operation on deviant trials (see Results for a better understanding of the rationale behind this normalization).

Before the taking part in the full experiment the participants were presented with a number of noise-vocoded speech snippets for ∼10 min. The goal of this was to enable subjects to become familiar with the peculiarity of noise-vocoded speech without allowing so much exposure as to enable substantial perceptual learning to take place (Sohoglu and Davis, 2016).

### Stimulus characterization

This study builds on a framework recently introduced by Di Liberto et al. (2015) that uses forward encoding models to predict EEG responses to natural speech. More specifically, it seeks to model how EEG responses vary as a function of particular features of the speech stimulus that are theorized to map onto different hierarchical levels of speech processing in the brain. To this end, three representations of the speech stimuli were used:

1. The spectrogram (S) was obtained by partitioning the speech signal into three frequency bands logarithmically spaced between 70 and 5000 Hz according to Greenwood's equation (70–494–1680–5000 Hz, the same used for the vocoder; Greenwood, 1961), and computing the amplitude envelope for each band, which was calculated as$\text{Env = }\left({\text{x}}_{\text{a}}\left(\text{t}\right)\right){\text{, x}}_{\text{a}}\left(\text{t}\right)\text{ = x}\left(\text{t}\right)\text{ + j}\hat{x}\left(\text{t}\right)$, where ${\text{x}}_{\text{a}}(\text{t})$ is the complex analytic signal obtained by the sum of the original speech x(t) and its Hilbert transform $\hat{x}(\text{t})\text{.}$
2. The phonetic-features (F) representation was computed using the Prosodylab-Aligner software (Gorman et al., 2011), which, given the speech file and its orthographic transcription, automatically partitions each word into phonemes from the American English International Phonetic Alphabet (IPA) and performs forced-alignment, returning the starting and ending time points for each phoneme. Each phoneme was then mapped to a corresponding set of 18 phonetic features, which was based on the University of Iowa’s phonetics project. In particular, the chosen features are related to the manner of articulation (plosive, fricative, nasal, liquid, and glide), to the place of articulation (bilabial, labio-dental, lingua-dental, lingua-alveolar, lingua-palatal, lingua-velar, and glottal), to the voicing of a consonant (voiced and voiceless), and to the backness of a vowel (front, central, and back). Also, a specific feature was reserved for diphthongs. As a result, this procedure produced a multivariate time series composed of 18 phonetic features, which describe specific articulatory and acoustic properties of the speech phonetic content.
3. Finally, we built a representation that combined F and S (FS) by applying a concatenation of the two representations. The idea of this combined representation is that the above spectrogram and phonetic feature representations are highly mutually redundant. This is because, on average, each phoneme will have a particular characteristic spectrotemporal profile. So if each phoneme were always spoken in the same way, then the two representations would be equivalent. However, in natural speech this is not the case, with significant variation in the spectrotemporal profile of a given phoneme across instances. So one might thus expect that an EEG encoding model based on categorical phonetic features (F), which is ignorant of these variations, would underperform relative to the abovementioned S model. However, it is also true that human listeners categorically perceive phonemes despite spectrotemporal variations, a fact that is presumably underpinned by consistent neural responses to those phonemes (Okada et al., 2010; Peelle et al., 2010). Such consistent responses would be captured by our F model and underrepresented by our S model because the latter is ignorant of the categorical nature of these utterances. As such, we contend that an EEG encoding model based on the concatenated representation, FS, should capture responses to both variable low-level acoustic fluctuations and categorical higher-level phonetic features.

Based on the above three representations, we have also previously suggested that one can attempt to isolate the unique contribution that derives from phonetic-feature level processing by subtracting the performance of the S model from that of the FS model (i.e., FS–S; Di Liberto et al., 2015; Di Liberto and Lalor, 2017).

A couple of final notes on our stimulus representations. Below, we also used a univariate envelope representation of the speech (E) for visualization purposes. This was calculated as the sum of the three band-limited envelopes that compose the S representation. In previous work, our framework has also included a phonemic representation of the speech (a multivariate time series of forced aligned phonemes, similar to F; Di Liberto and Lalor, 2016). However, because of the limited amount of speech data used in the present study, less frequent phonemes would not have a sufficient number of occurrences to produce a good model fit. As a result, we did not include this representation in the present study and focused our analysis on the more fundamental phonetic-features model. As an aside, if it were of interest, the scalp responses to phonemes can still be visualized by performing a linear projection of the F model (in fact, a phoneme can be represented as a combination of specific phonetic features).

### EEG data analysis

The EEG signals were analyzed offline using MATLAB software. Because of suggestions that speech tracking in the δ-band (1–4 Hz) and θ-band (4–8 Hz) might have different functional roles in speech processing (Ding and Simon, 2014), we analyzed these two EEG bands separately. Specifically, the data were digitally filtered into the two frequency bands of interest using Chebyshev type-2 bandpass filters with pass-band between 1 and 4 Hz (δ-band) and between 4 and 8 Hz (θ-band). Next, signals were down-sampled to 128 Hz, and referenced to the average of the two mastoid channels. EEG channels whose time-series data had a variance that exceeded three times that of the surrounding channels were identified as being excessively noisy. And the data on those channels were replaced by spline interpolating the data from the surrounding clean channels using EEGLAB software (Delorme and Makeig, 2004).

Linear regression was used to create a mapping between the EEG and the abovementioned three speech stimulus representations (Fig. 1*B*). For each representation, the result of the linear regression consists of a set of weights referred to as a multivariate temporal response function (TRFs; Crosse et al., 2016). A multivariate TRF (mTRF) can be interpreted as a filter that describes the brain’s linear mapping of a continuous stimulus feature, *S*(*t*), to the corresponding continuous neural response *R*(*t*), i.e.,

R(t)=mTRF*S(t),

where * represents the convolution operator. The mTRFs were calculated by performing ridge regression between the stimulus features and the corresponding EEG. This approach allows for the use of a regularization parameter (*λ*), which can improve the quality of fit (in the case of noisy data) and controls overfitting by assuming a certain level of temporal smoothness (Crosse et al., 2016b).

Speech stimuli and the corresponding EEG responses were partitioned into 10 equal-sized subsets *S_1_*, *S_2_*, …, *S_10_*, and *R_1_*, *R_2_*, …, *R_10_*, respectively; *k*-fold cross-validation (*k* = 10) was employed on these partitions to compare how each speech representation (S, F, and FS) mapped to the EEG. In particular, EEG signals of a subset *i* (*R_i_*) were predicted using models that were fit to each distinct speech representation on all the left-out partitions (1,…,*i*-1,*i* + 1,…,10), and prediction accuracies were quantified for each electrode using a Pearson correlation. To optimize performances, we conducted a parameter search (over the range 10^−3^, 10^−2^, …, 10^5^) for the regularization parameter *λ* within each speech representation model. This procedure maximized the EEG prediction accuracy averaged across trials, subjects, and all 128 electrodes. The combination of regularization and cross-validation controlled for overfitting and prevented bias toward the test data used for quantifying the prediction accuracies.

The mTRF mapping from speech to EEG signals is sensitive to the selection of both a temporal window and an electrode set of interest. The time window specifies which time lags between speech and EEG are considered for the model fit. The basic rationale is that an unpredictable stimulus (delivered at time lag zero) induces a cortical response that begins after lag zero and may continue for a certain length of time, which is on the order of hundreds of milliseconds and depends on the complexity of the related cortical process. For this purpose, a time-lag window between −50 and 250 ms was selected, as it produced the best EEG prediction accuracies for clear speech. After the time-lag window selection and λ optimization, a set of 12 consistently well-predicted electrodes (six on the left side of the scalp and their symmetrical counterparts on the right; Di Liberto et al., 2015) from fronto-temporal regions of the scalp were selected for calculating the EEG prediction accuracies.

This procedure resulted in EEG prediction measures for all the speech representations described in Stimulus characterization above. And, as mentioned above, an additional quantitative measure was derived that accounted for the unique gain in predictability provided by the use of phonetic features, compared to when only spectral features were used, i.e., FS–S (Di Liberto et al., 2015; Di Liberto and Lalor, 2017).

### Statistical analysis

Statistical analyses were performed using a repeated measures ANOVA to compare distributions of Pearson correlation values across models. ANOVA analyses were conducted after verifying that the normality assumption was not violated, which was assessed both visually (QQ plots; data not shown) and quantitatively (Shapiro–Wilk test). The values reported use the convention *F*(*df*, *dferror*). Greenhouse-Geisser corrected degrees of freedom are reported where the assumption of sphericity was not met (as indicated by a significant Mauchly’s test). All *post hoc* model comparisons were performed using Bonferroni-corrected paired *t* tests. Two-tailed permutation tests with 200,000 repetitions were used for pair-wise comparisons if the assumption of normality was violated (Shapiro–Wilk test). While it is customary to apply Fisher’s *z*-transformation to Pearson correlation scores before performing statistical analysis on those scores, we did not do that for the results presented below. The rationale for the Fisher transform is to normalize the sampling distribution of the (usually skewed) Pearson’s *r* values and to produce a less biased statistic. However, in our case, the *r* values are really quite low and are, generally speaking, already normally distributed. And it has been suggested that with large numbers of data points and small *r* values, applying a Fisher’s *z*-transformation can in fact lead to a more biased result (Corey et al., 1998). (Incidentally, despite our concerns that Fisher transforming our data may produce a larger bias, we ran the same set of analyses on both the raw *r* values and the Fisher transformed values. No qualitative differences were observed, so we only present the results from the raw *r* values for the abovementioned reasons.) Effect size is reported for both *t* test and ANOVA analyses. Specifically, Cohen’s effect size absolute value (|*d|*) is reported for *t* test and partial eta-squared (*η* ^2^) is used for ANOVA. Linear mixed-effects models were fit using the maximum likelihood criterion and Satterthwaite approximation was used for computing the denominator degrees of freedom for the F statistics reported.

## Results

### P enhances perceived speech clarity

**Figure 2.**
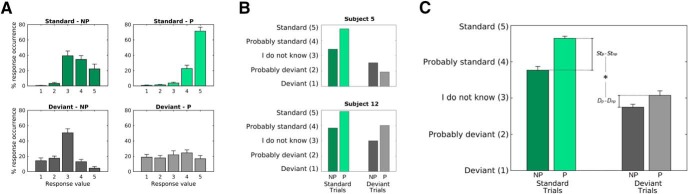
A behavioral measure of speech clarity reflects the effect of P. Subjects were presented with sequences of vocoded-original-vocoded speech snippets and were asked to identify the two noise-vocoded streams (NP and P stimuli) as standard or deviant presentations by comparing them with the original speech snippet. Responses consisted of a level of confidence from 1 (definitely a deviant) to 5 (definitely a standard). ***A***, The response distributions (mean percentage occurrence ± SEM) confirm that subjects were more confident in detecting standard trials when P was available. ***B***, The confidence level for two selected subjects. The result in the top panel shows that subject 5 improved in detecting both standard and deviant trials when P was available, which we interpret as evidence for an increase in perceptual clarity. In contrast, subject 12 (bottom panel) responded with higher values to P stimuli for both standard and deviant trials. In this case, the positive St_P_-St_NP_ cannot be assumed to purely reflect an increase in perceived clarity, as deviants were not detected. ***C***, The confidence level averaged across all subjects (mean ± SEM) is here reported for NP and P stimuli, and for both standard and deviant trials. The increase in confidence due to P is larger for standard than for deviant trials (*p < 0.05).

Participants were asked to identify the first (NP) and the second (P) speech vocoded streams as a standard (*St*) or deviant (*D*) presentation using a level of confidence from 1-5 (from ‘definitely a deviant’ to ‘definitely a standard’ respectively). The response distribution for each condition (averaged across subjects; Fig. 2*A*) indicates that participants were more confident in identifying standard trials when P was available (standard P compared to standard NP), while this was not the case for deviant trials (deviant P compared to deviant NP). Note that subjects were instructed to report detection of a deviant trial only if they heard a difference with the corresponding clear speech snippet. But because perceptual pop-out did not occur for the modified portion of the *D_P_* trials, this was a more difficult determination for subjects to make. For this reason, P improved the standard but not the deviant detection scores.

A significant enhancement of the detection score from NP to P was observed for standard trials (*St_P_* > *St_NP_*, permutation test, *p* = 0.001), which confirms that P had an effect on subjects’ confidence in detecting standard trials. However, this alone is not sufficient to draw conclusions about the effects of P on the perceived speech clarity. This is because it was possible that subjects may have been biased to respond to both standard and deviant stimuli as standard trials when prior information was available. For example, this was the case for subject 12, whose individual behavioral scores are reported in Figure 2*B*, bottom panel. In contrast, subject 5 (Fig. 2*B*, top) exhibited an increase of speech clarity with P, as detection for both standard and deviant improved for P trials. to control for such biases across individual subjects, a subject-specific baseline was derived using deviant trials and subtracted from the confidence level for standard trials. This corrected behavioral measure (*St* − *D*) exhibited a significant interaction with P (*St_P_* − *St_NP_* > *D_P_* − *D_NP_*, permutation test, *p* = 10^−6^). This result, which is depicted in Figure 2*C*, indicates an increase in perceived speech clarity due to P of the upcoming stimulus. This perceptual enhancement can be summarized for each single subject using the following quantitative measure:

$$\Delta_{Clarity}=(ST_{P}-ST_{NP})-(D_{P}-D_{NP}).$$

Interestingly, the result in Figure 2 shows that the NP vocoded speech snippets, although severely degraded, were perceived as partially intelligible rather than completely unintelligible (*StNP* > *DNP*, permutation test, *p* = 10^−6^). These results indicate that, as hypothesized, prior information led to clearer perception of the noise-vocoded speech stimuli, a perceptual difference that we have quantified as $\Delta_{\text{Clarity}}$.

### Dual effect of P on the cortical entrainment to speech features

**Figure 3.**
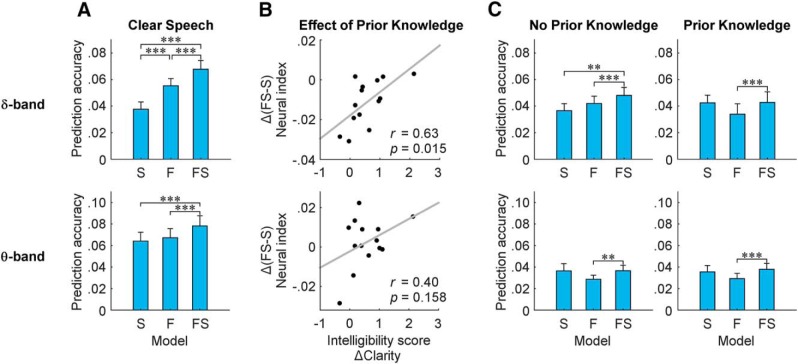
The effect of P on EEG predictability. Linear regression was used to fit models known as multivariate temporal response functions (mTRFs) between the low-frequency (δ-band: 1–4 Hz and θ-band: 4–8 Hz) EEG and different representations of the speech stimulus. In particular, speech was represented as its spectrogram (S), a time-aligned sequence of categorical phonetic features (F), or a combination of both (FS; *p ≤ 0.05, **p ≤ 0.01, ***p ≤ 0.001). The difference in performance between the FS and S models (i.e., FS–S) is taken as an isolated measure of phoneme-level encoding. ***A***, Correlations (mean ± SEM) between recorded EEG and EEG predicted using the mTRF models for spectrogram (S), phonetic features (F), and their combination (FS) for clear speech. ***B***, A significant positive correlation emerges between the change in perceived intelligibility (measured as Δclarity) and the change in our isolated index of phoneme level δ-band entrainment from NP to P speech segments ((FS–S)_P_ – (FS–S)_NP_) as a result of P. ***C***, Correlations (mean ± SEM) between recorded EEG and EEG predicted using the mTRF models for spectrogram (S), phonetic features (F), and their combination (FS) for noise-vocoded speech. In the δ-band, the FS model performs best for the NP speech segments (NP) but not for the P segments (P). No significant differences emerge in the θ-band.

EEG predictability measures were derived using a forward mTRF model that estimates an optimal linear mapping from a speech representation to the corresponding scalp-recorded EEG signal. These predictability measures were derived for different frequency bands (delta and theta) and models (S, F, and FS). A significant interaction between these two factors emerged from a unified 2 × 3 ANOVA analysis for the C and NP conditions, but not for P (two-way ANOVA, C: *F*(1.37,17.85) = 6.261, *p* = 0.015, effect size = 0.33; NP: *F*_(1.19,15.48)_ = 8.454, *p* = 0.008, effect size = 0.39; P: *F*_(1.26,16.42)_ = 0.233, *p* = 0.692, effect size = 0.018). Based on this interaction, follow-up one-way ANOVAs were conducted for the δ-band (1–4 Hz) and θ-band (4–8 Hz) separately and the results were compared between the NP, C, and P stimuli. In the δ-band, the analysis for C stimuli (Fig. 3*A*, top) showed that the combined FS model performed better than both S and F models, and that the F model performed better than the S model (ANOVA: *F*_(1.41,19.70)_ = 48.226, *p* = 1.7 × 10^−7^, effect size = 0.763; *post hoc* paired *t* test comparisons: *p* = 10^−6^, *p* = 3.5 × 10^−5^, *p* = 9 × 10^−4^ for S vs FS, F vs FS, and S vs F, respectively). Furthermore, the analysis for C stimuli in the θ-band (Fig. 3*A*, bottom) showed that the combined FS model performed better than both S and F models; however, no significant difference emerged between the F model and the S model (ANOVA: *F*_(1.26,16.37)_ = 14.490, *p* = 8.5 × 10^−4^, effect size = 0.527; *post hoc* paired *t* test comparisons: *p* = 0.002, *p* = 5 × 10^−6^, *p* = 1 for S vs FS, F vs FS, and S vs F respectively). These results are consistent with those obtained previously for clear natural speech using a different dataset (Di Liberto et al., 2015).

As mentioned above, and in our previous studies, we have suggested that isolated indices of speech-specific processing can be quantified using our analysis framework. In particular, as depicted in Figure 1*B*, we suggest that this can be done by noting that the FS model is sensitive to activity reflecting the processing of both sound acoustics and categorical phonetic features, while the S model does not explicitly encode phonetic features and should thus be less sensitive to the categorical processing of those features (Di Liberto et al., 2015). Therefore, we propose that any difference in EEG prediction accuracy between the two models would be due to the fact that the FS model captures extra activity reflecting the processing of categorical phonetic features. And, as such, we suggest that one can isolate a measure of speech-specific cortical processing at this level by subtracting ${\text{r}}_{\text{S}}$ from ${\text{r}}_{\text{FS}}$ (i.e., FS–S). Here, we hypothesized that this measure would be particularly sensitive to differences in perceived clarity as a result of P. Specifically, our hypothesis was that, because the perceived speech clarity (and therefore intelligibility) of the two conditions differed as a result of P, we would see a clear increase in our proposed isolated measure of phonetic feature-level processing (FS–S) with P. In line with other work (Holdgraf et al., 2016), we also wished to explore the possibility that top-down effects on the processing of speech may impact even earlier stages of speech encoding at the level of acoustics, as indexed via the S model. The effect of P on the FS–S measure was quantified as:

$$\Delta (FS-S)=(r_{FS}-r_{S}{)}_{P}-(r_{FS}-r_{S}{)}_{NP}.$$

In line with our primary hypothesis, we found that Δ(FS–S) in the δ-band was positively correlated with the behavioral measure $\Delta_{\text{Clarity}}$ across subjects (Fig. 3*B*). That is to say, the larger the enhancement in speech clarity due to prior information for a given subject, the bigger Δ(FS–S) for that subject (Pearson’s correlation coefficient *r* = 0.63, *p* = 0.015). Somewhat surprisingly, no such correlation emerged for θ-band Δ(FS–S) (Pearson’s correlation coefficient *r* = 0.40, *p* = 0.158). This result suggests that the δ-band neural measure FS–S, which we take as in index of phonetic-feature encoding, is sensitive to increases in the perceived clarity of speech that come with access to P.

An additional statistical analysis was conducted to exclude possible effects of subject variability due to noise. This was a possibility because the neuro-behavioral correlation shown in Figure 3*B* is the result of a between-subject analysis. This confound was excluded by means of a linear mixed-effects analysis that accounts for both inter-trial and inter-subject variability. Our speech-specific neural index (FS–S) was the continuous numeric dependent variable and P (P vs NP) was a continuous numeric fixed factor. Between-subject and between-trial variation were accounted for as random effects. We found a significant main effect of P on FS–S (*p* = 0.034) and on the behavioral measures (*p* = 1.6 × 10^−214^). Interestingly, however, for a majority of subjects (11 out of 14), and despite the positive correlation with behavior, our neural index of phoneme level processing (FS–S) actually decreased with prior information, a finding that ran counter to our primary hypothesis. This suggests the possibility of a second effect involving a suppression of responses at this hierarchical processing level to the P condition relative to NP (*t* test on FS–S: *p* = 0.003, effect size = 0.863).

To clarify the factors that led to the suppressive effect of P on the δ-band cortical index FS–S, the various model performances were compared for the NP and P stimuli. It is important to re-emphasize that each pair of NP and P stimuli had identical physical properties. Therefore, significant differences in the corresponding scalp responses must be due to some combination of the following two factors: (1) it could be related to the enhancement of perceived clarity with prior information, a suggestion that is supported by our abovementioned positive correlation between Δ_Clarity_ and Δ(FS–S), and (2) it could be related to the fact that the P stimulus is a repetition of a previously presented stimulus, while the NP stimulus is always a first presentation. If the latter is a factor in causing a reduction in δ-band EEG prediction accuracy, it should be evident in the pattern of model performances, although it would still remain to explain precisely what mechanisms underlie such effects (e.g., predictive coding vs adaptation – see discussion). Indeed, results for the NP and P stimuli exhibited different patterns in terms of the relative model performances (Fig. 3*C*). Specifically, the model performances for NP were similar to those for clear speech, with the combined FS model performing better than both S and F (ANOVA: *F*_(1.14,14.87)_ = 7.22, *p* = 0.014, effect size = 0.357; *post hoc* paired *t* test comparisons of FS with all other models: *p* = 0.012, *p* = 0.001 for S and F respectively). This was not the case for the responses to the P stimuli. In fact FS performed better only than F, while no significant difference emerged when compared with S (ANOVA: *F*_(1.29,16.72)_ = 4.24, *p* = 0.040, effect size = 0.246; *post hoc* paired *t test* comparisons of FS with all other models: *p* = 1, *p* = 0.001 for S and F respectively). The model predictions were generally lower for NP stimuli than for clean speech (paired *t test* on S: *p* = 0.88, effect size = 0.056; F: *p* = 0.04, effect size = 0.658; FS: *p* = 0.01, effect size = 0.832), but had a similar relative performance pattern between models, which was not particularly surprising given that noise vocoding reduced the intelligibility of the NP stimuli, but did not make them completely unintelligible.

This pattern of results suggests that the δ-band EEG predictability measures are sensitive to the effect of P, and that this P primarily affected the interaction between acoustic (S) and phonetic (F) speech models, rather than any individual model performance. In fact, no significant effect (enhancement nor suppression) emerged for any single speech representation/model between NP and P (paired *t test* on S: *p* = 0.16, effect size = 0.287; F: *p* = 0.16, effect size = 0.317; FS: *p* = 0.29, effect size = 0.200). Unlike in the δ-band, EEG predictability in the θ-band did not exhibit different results patterns for NP and P stimuli. Importantly, no significant difference emerged between FS and S for either NP or P stimuli, suggesting that cortical entrainment measures in the θ-band are not affected by differences in perceived clarity (NP stimuli: ANOVA, *F*_(1.17,15.16)_ = 4.83, *p* = 0.039, effect size = 0.271; *post hoc* paired *t* test comparisons: ***p* = 1**, *p* = 0.002, *p* = 0.208 **for S vs FS**, F vs FS, and S vs F, respectively; P stimuli: ANOVA, *F*_(1.09,14.22)_ = 5.97, *p* = 0.026, effect size = 0.314; *post hoc* paired *t* test comparisons: ***p* = 1**, *p* = 4.3 × 10^−5^, *p* = 0.292 **for S vs FS**, F vs FS, and S vs F, respectively).

### Differential effects of P on distinct phonetic features

**Figure 4.**
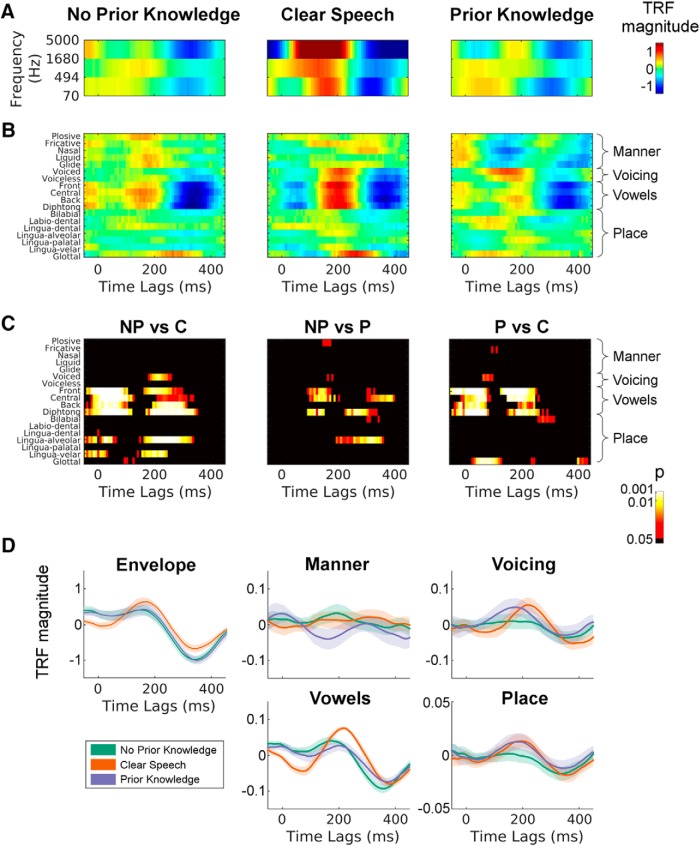
The effect of P on the TRFs. ***A***, The TRF (model weights) for the spectrogram representation of speech (S) are shown for all conditions after averaging across 12 selected electrodes (see Materials and Methods, EEG data analysis). To allow a direct comparison of all conditions, the TRF for the C model is shown using only three frequency bands, although the model used in the analysis included all 16 bands. Colors indicate the TRF magnitude (arbitrary units). ***B***, TRF models fit using phonetic features (F) are shown for all conditions. ***C***, F model weights were compared between each pair of conditions using *t* tests at each time lag and phonetic feature. ***D***, To more directly compare the TRF weights between conditions, univariate models are shown for the envelope of speech and for four distinct groups of phonetic features (average weights of each group are reported): manner of articulation, voicing, vowels, and place of articulation.

The results so far suggest that P affects the EEG-measured cortical tracking of speech and, crucially, the correlation between perceived clarity and FS–S links this effect directly with the cortical processing of phonetic features of speech. To examine how prior information affects specific speech features, we compared the model-weights across conditions, speech representations, and time lags in the δ-band (Fig. 4). It is important to note that the advantages of using EEG prediction accuracy as a dependent measure are that (1) it can combine information across features and frequency bands into one optimal prediction and (2) it produces a long vector in the time domain that, despite its low SNR, produces robust and reliable correlations with the actual EEG. Analyzing the TRF weights over different features typically involves dealing with a lot of variability, at least with the amount of data in the present study. Nonetheless, we conducted this analysis on a time lag window of −100 − 500 ms, which allowed for a clearer contrast between more and less meaningful time lags. In addition, the TRF-weights shown in the figure were averaged across a set of 12 fronto-central well-predicted electrodes.

Unfortunately, it is not straightforward to examine the model weights of FS–S itself, given that these two models correspond to feature-spaces with different dimensionality. However, one can still seek some extra insight by separately examining the weights of the acoustic and phonetic models. The acoustic models, which were fit using the envelope and the 3-band spectrogram of speech, showed similar weights for NP and P, while there were stronger average responses in the C condition compared to NP and P, although these differences were not significant (Fig. 4*A*). A more interesting pattern of results emerged for the F model (Fig. 4*B*). In particular, there appeared to be differences between the C, P, and NP models in the vowel-based features of the TRF (Fig. 4*B*). These differences were supported by a simple exploratory statistical cluster analysis that compared the phonetic feature TRFs between conditions (uncorrected *t* tests at every time lag and for every feature; Fig. 4*C*). While there were some time points that also showed differences between NP and P, these effects were not very robust and did not survive correction for multiple comparisons. To examine this in another way, we collapsed the TRFs across phonetic-feature categories (Manner of Articulation, Voicing, Vowels, and Place of Articulation) and examined the resulting one-dimensional TRFs across conditions (along with the standard Envelope TRF for comparison; Fig. 4*D*). A significant suppression of the N1_TRF_ and P1_TRF_ components for vowel features emerged for NP and P compared with C (permutation test between NP and C models: *p* < 0.05 for −15–85 ms and 195–312 ms; permutation test between P and C models: *p* < 0.05 for −15–54 ms and 187–250 ms; significant clusters with more than two contiguous time lags were excluded; Fig. 4*D*). Interestingly, although not significant, the average suppression was greater for P compared to NP. Qualitatively, consonant voicing and place of articulation features resemble the weights for clear speech in the P but not in the NP condition, while no obvious similarity across conditions emerged for manner of articulation features, although there were no statistically significant effects on this.

## Discussion

This study investigated the effect of P on the cortical tracking of acoustic and phonetic speech features using non-invasive EEG and an analysis framework based on ridge regression and EEG predictability (Di Liberto et al., 2015; Crosse et al., 2016). The results observed for the clear speech reproduced the ones shown previously by Di Liberto et al. (2015). In the δ-band, a weaker but similar pattern emerged for NP stimuli, which were only partially intelligible because of a severe degradation of their acoustic properties. Crucially, a different results pattern was observed for P stimuli, indicating that P modulates the cortical entrainment to speech features. We hypothesized that this phenomenon would be reflected in an increase in a novel measure of cortical entrainment to speech-specific phonetic features (FS–S). This hypothesis turned out to be partially supported by our data, which exhibited two top-down effects of P. The first effect was in line with our hypothesis and took the form of a positive correlation between our neural measure and perceived clarity across subjects. The second, *post hoc* effect, ran counter to our hypothesis and took the form of an overall reduction in EEG prediction accuracy for the P stimuli.

Previous research has failed to find any effect of perceived speech intelligibility on low-frequency cortical tracking of the speech envelope using a perceptual pop-out task (Millman et al., 2015; Baltzell et al., 2017). This is consistent with our findings in that we saw no correlation between perceived clarity and tracking of low-level acoustics (via the S model). It was only by using differential model performances as our index (FS–S) that we were able to isolate processing at the phonetic-feature level and reveal a relationship. This points to a concern about relying on envelope tracking as a measure of speech processing (Obleser et al., 2012). Specifically, it is highly likely that such a reliance leads to neural indices that reflect multiple, distinct functional processes (Ding and Simon, 2014), making it difficult to determine to what extent the indices reflect speech-specific activity. This might explain why there has been a lack of consistency across studies aimed at examining the effects of speech intelligibility on neural measures of envelope tracking (Howard and Poeppel, 2010; Peelle et al., 2013; Ding et al., 2014). We suggest that our approach may represent one way of partially disentangling the multiple processes that must be active during natural speech perception.

The idea that our approach could allow us to distinguish between different levels of hierarchical processing may also explain the apparent contrast between our results and recent ECoG work showing changes in spectrotemporal tuning in auditory cortex using a very similar paradigm (Holdgraf et al., 2016). The results of that study might suggest that we should have seen changes in our S model performance as a function of P, something that we did not observe. While we originally hypothesized that our paradigm should lead to the strongest effects at the phonetic-feature level, there is no obvious reason why top-down information could not penetrate further down the hierarchy to affect the acoustic encoding of speech. So why do we not see it in the S model? There are several possible reasons. It may be that there is a dissociation between the information carried by high-γ in the ECoG data (Holdgraf et al., 2016) and by our low-frequency EEG. Or it may be that the lower SNR of EEG makes it difficult to see what may only be subtle effects in the S model. Another possibility, though, is that the spectrotemporal tuning changes in the superior temporal gyrus (STG) reported by Holdgraf et al. (2016), may actually reflect changes in the encoding of categorical phonetic features. As we discuss above, there is undoubtedly a lot of redundancy between acoustic and phonetic-feature representations. But also it has been suggested that STG may be a transitional stage, early enough to still encode acoustic features of speech but high enough to exhibit response selectivity to feature combinations and encoding of categories (Mesgarani et al., 2014; Shamma, 2014). So, while we cannot be conclusive on this point, it may be the case that our approach has allowed for a finer-grained analysis in terms of the hierarchical stages that are affected by prior information.

While our results indicate that P affects the cortical encoding of speech-specific features, it remains unclear how this effect comes about. One possibility is that top-down prior information directly impacts lower-level sensory processing at the acoustic and phonetic encoding stages, leading to enhanced perceptual clarity. This interpretation is in line with ECoG recordings in STG that showed that phonemic restoration of missing speech can be predicted by specific neural activity patterns (Leonard et al., 2016). Another possibility is that our effects may be more indirectly driven by increases in attention due to the perceptual enhancement. Future work will aim to examine this by adding controlled attentional manipulations and by quantifying the causal impact of frontal signals on our auditory cortical measures, as has been done for envelope tracking (Park et al., 2015) and event-related responses (Sohoglu et al., 2012).

The effects of P discussed here emerged only in the δ-band of the EEG. This is in line with a current view suggesting that δ- and high-frequency activity (>40 Hz) are reliable indicators of perceived linguistic representations, while θ-band activity may primarily reflect the analysis of the acoustic features of speech (Kösem and van Wassenhove, 2016). Indeed one study, in particular, examined the cortical tracking of vocoded speech in background noise and found that δ-band tracking correlated with speech recognition scores across subjects (Ding et al., 2014), a result that corresponds very nicely with our neural-behavioral correlation. However, the specificity of our effects to the δ-band also appears to run counter to other studies examining the relationship between cortical tracking of vocoded speech and intelligibility (Peelle et al., 2013). That study reported significant differences between the cortical tracking of intelligible and unintelligible (vocoded) speech in the θ-band. That said, the authors of that study reported no correlation between their behavioral measures of intelligibility and their θ-band tracking indices. In addition, they did not control for the fact that their intelligibility manipulation (vocoding) covaried with the amount of sensory detail in their stimuli, an issue that we have attempted to address and that has been shown to be important in their more recent work (Blank and Davis, 2016). So it is possible that their θ-band effects actually reflect something other than intelligibility and, therefore, that they do not in fact conflict with our findings. Future work including intelligibility manipulation with multiple levels of strength will be needed to more directly compare our finding with the current literature.

Our results suggest the emergence of two effects of perceptual pop-out. This is consistent with previous studies suggesting that P may produce counteracting effects (e.g., Tuennerhoff and Noppeney, 2016). One view is that predictions increase the perceived clarity by inducing a better synchronization of the cortical responses to speech (Peelle et al., 2013), which would produce larger cortical entrainment measures. Along the same lines, it has been proposed that increased entrainment measures may reflect the activation of higher-order areas that would have been “inactive” or less responsive when perceived clarity was degraded (Davis and Johnsrude, 2003; Peelle and Davis, 2012; Tuennerhoff and Noppeney, 2016). Both of these ideas are consistent with our positive neural-behavioral correlation across subjects. On the other hand, predictive coding theories assert that P of an upcoming stimulus should suppress the measured cortical responses, as those responses are proposed to represent the error between what is predicted and the bottom-up sensory input (Friston, 2005; Clark, 2013). And this would be consistent with the overall suppression we see in our neural index of phonetic-feature encoding.

While the neural-behavioral correlation we report was in line with our initial hypothesis, we did not anticipate the overall suppression of the neural index FS–S. However, the latter result is consistent with the late suppression in left STG shown by Sohoglu et al. (2012) and in line with predictive coding theories. Indeed, because of our experimental design, the stimulus repetition for P trials may contribute to this suppressive phenomenon. On the one hand, it has been hypothesized that such suppressive effects are automatic and due to stimulus-induced neural adaptation (Grill-Spector et al., 2006). On the other hand, the suppression may be a consequence of top-down predictions and could be explained via the theory of predictive coding (Summerfield et al., 2008; Todorovic et al., 2011). Research on repetition suppression usually involves short, isolated auditory stimuli (e.g., tones), which are very different from the 10-s sentences used in the present study. As such, we are inclined to tentatively suggest that repetition suppression and adaptation will not have played a major role in our findings, but rather that our suppression effects are likely a consequence of predictive coding. Indeed a review of predictive coding theory has proposed that there may exist two distinct units within our sensory processing hierarchies: representational/state units and error units (Friston, 2010; Hohwy, 2013). And this idea fits well with our dual effects. It may be the case that activity from representational units in deeper cortical layers is increased with P in our experiment, while activity from error units in more superficial layers is suppressed. Future work involving a more balanced factorial design may be able to more clearly separate these two effects. In particular, it would be interesting to manipulate both the strength and validity of predictions, and the level of speech degradation, so as to be able to disentangle the effects of prediction and prediction error on our tracking measures. This type of design has been used before to show, not only changes in evoked activity, which is what likely what our δ/θ predictions are capturing, but also how those changes relate to beta and gamma oscillations within a discrete, multisensory speech paradigm (Arnal et al., 2011). The ensuing results supported the notion that beta activity reflects top-down predictions, while gamma power carries information about prediction errors. In the context of continuous speech, it would be very interesting to see if the relationship between our evoked tracking measures and oscillatory activity fluctuates as a function of the strength and validity of predictions, and to examine any such relationship using source-localized connectivity approaches and/or dynamic causal modeling (Friston et al., 2003). We examined the model weights of the various TRFs in an effort to determine what specific processes might be driving our EEG prediction accuracy effects (Fig. 4). The most notable finding was that there appeared to be differences between the C, P, and NP models in the vowel-based features of the F model TRF. We think this makes good sense when comparing the C condition with the two vocoded conditions as vowels are primarily defined by their spectral content, which is what is lost by noise vocoding. But, importantly, a small number of time points showed differences in vowel-related activity between NP and P, which may reflect some kind restoration of vowel processing with prior information in the P condition. We intuitively feel that the restoration of vowel processing with prior information makes sense given the nature of the information lost in noise vocoding. That said, these effects were not robust to correction for multiple comparisons as they showed a high-degree of variability across subjects. This, combined with the likely counteracting effects of increased clarity and reduced prediction error make it impossible for us to be too definitive on this point. Finally, we saw interesting qualitative similarities between the TRFs for “place of articulation” and “voicing” between the P and C conditions, suggesting that these may also be interesting targets for future research in terms of which features are restored with prior information.

In summary, we contend that the present work provides an isolated quantitative measure of the cortical encoding of speech-specific features. This measure, here referred to as FS–S, was shown to correlate with the behaviorally-measured perceived clarity of degraded speech. We previously suggested that this measure might index the cortical encoding of phonetic features, which has formerly been associated with the STS (Hickok and Poeppel, 2007; Overath et al., 2015). And, interestingly, a recent fMRI study has pointed to a specific role for the STS in underpinning the improved perception of degraded speech that comes about with P (Blank and Davis, 2016). In particular, multivariate BOLD analysis showed interacting effects of sensory detail and prior information in STS. While it is difficult to definitively relate these effects to our study, the fact that our data suggests the possibility of two counteracting mechanisms (overall suppression and between-subject increase of FS–S), leads us to speculate that the FS–S index reflects activity, at least partially, from STS. In the opposite direction it also provides a link between those fMRI findings and the low-frequency cortical entrainment phenomenon.

## Synthesis

Reviewing Editor: Satu Palva, University of Helsinki Decisions are customarily a result of the Reviewing Editor and the peer reviewers coming together and discussing their recommendations until a consensus is reached. When revisions are invited, a fact-based synthesis statement explaining their decision and outlining what is needed to prepare a revision will be listed below. Note: If this manuscript was transferred from JNeurosci and a decision was made to accept the manuscript without peer review, a brief statement to this effect will instead be what is listed below.

I have looked over the rebuttal letter that accompanied this revised manuscript, and it is my assessment that you have satisfactorily addressed all the concern raised by the reviewers who previously reviewed this manuscript. Therefore, it's my judgment that your manuscript does not require further peer review before being accepted for publication in eNeuro.
